# Barriers to childhood immunization in sub-Saharan Africa: A systematic review

**DOI:** 10.1186/s12889-020-09169-4

**Published:** 2020-07-14

**Authors:** Joseph Benjamin Bangura, Shuiyuan Xiao, Dan Qiu, Feiyun Ouyang, Lei Chen

**Affiliations:** 1 Department of Epidemiology and Health Statistics, Xiangya School of Public Health, Central South University, Changsha, China; 2 Department of Social Medicine and Health Management, Xiangya School of Public Health, Central South University, Changsha, China; 3 Department of Pediatrics, Faculty, Global Health Initiative, Yale University School of Medicine, New Haven, USA

**Keywords:** Barriers, Childhood, Immunization, Sub-Saharan Africa

## Abstract

**Background:**

Immunization to prevent infectious diseases is a core strategy to improve childhood health as well as survival. It remains a challenge for some African countries to attain the required childhood immunization coverage. We aim at identifying individual barriers confronting parents/caretakers, providers, and health systems that hinder childhood immunization coverage in Sub-Saharan Africa.

**Method:**

This systematic review searched PubMed/MEDLINE, Web of Science and EMBASE. We restricted to published articles in English that focused on childhood immunization barriers in sub-Saharan Africa from January 1988 to December 2019. We excluded studies if: focused on barriers to immunization for children in other regions of the world, studied adult immunization barriers; studies not available on the university library, they were editorial, reports, reviews, supplement, and bulletins. Study designs included were cross-sectional, second-hand data analysis; and case control.

**Results:**

Of the 2652 items identified, 48 met inclusion criteria. Parents/caretakers were the most common subjects. Nine articles were of moderate and 39 were of high methodological quality. Nine studies analyzed secondary data; 36 used cross-sectional designs and three employed case control method. Thirty studies reported national immunization coverage of key vaccines for children under one, eighteen did not. When reported, national immunization coverage of childhood vaccines is reported to be low. Parents/caretaker’ barriers included lack of knowledge of immunization, distance to access point, financial deprivation, lack of partners support, and distrust in vaccines and immunization programs. Other associated factors for low vaccine rates included the number of off-springs, lifestyle, migration, occupation and parent’s forgetfulness, inconvenient time and language barrier. Barriers at health system level cited by healthcare providers included limited human resources and inadequate infrastructures to maintain the cold chain and adequate supply of vaccines.

**Conclusion:**

In this review we identified more thoroughly the parents/caretakers’ barriers than those of providers and health systems. Factors that influenced decisions to get children vaccinated were mainly their gender, beliefs, socio-culture factors in the communities in which they live. Thus it is vital that immunization programs consider these barriers and address the people and societies in their communities across sub-Saharan Africa.

## Background

Immunization is a protective measure against infectious diseases [1]. Childhood immunization remains one of the highest impact public health interventions, reducing infectious diseases-related morbidly and mortality of children at a low cost [2]. It is a core child survival strategy and is demonstrated to avert more than 1·2 million child deaths each year [3, 4]. It is a key strategy towards attaining Sustainable Development Goal (SDG) number 3, namely the *reduction of under-five mortality to less than 25/1000 live births by 2013* [5]. Despite these gains, vaccine-preventable diseases remain a major cause of child illnesses and deaths, particularly in low-income countries [6].

Africa has the highest under-five mortality rate of the entire world and accounts for 40% of the total deaths in this age group. This is mainly due to vaccine-preventable diseases [7]. Over the past few decades, African immunization programs have made progress, yet coverages remains low for some recommended childhood vaccines. In 2014, it was reported that only Zimbabwe among the Sub-Saharan region was estimated to have met the Global Vaccine Action Plan threshold of 80% or higher coverage of diphtheria–tetanus-pertussis vaccine (DTP3), a benchmark used to measure performance of routine vaccine delivery system [8]. In 2016, one in five African children goes without lifesaving vaccines [9]. Most African countries are unable to reach the most vulnerable children populations in remote and rural communities [5, 10]. Studies [1–3, 7, 11–54] conducted in Africa have attempted to elucidate potential barriers that lead to low uptake and none-completion of immunization series. Previous review [55] exploring reasons related to non-vaccination and under-vaccination of children in low- and middle-income countries categorized factors into major themes: Immunization systems; communication and information; family characteristics and parental attitudes/knowledge. However, it noted the lack of peer reviewed literature in Central Africa. Another review [56] investigated factors associated with incomplete or delayed vaccination across countries. Despite its potential importance, it did not categorize findings into major domains, as policy implication for each might be different.

This systematic review aims at identifying relevant studies and summarizing major barriers confronting health systems, providers, and caregivers that hinder immunization coverage in sub-Saharan Africa. The results of this review will add to existing knowledge of the problem, and guide policy makers to improve immunization programs in sub-Saharan Africa, especially in those countries where the included studies had been conducted; and also to provide useful information for further research on these problems.

## Methods

### Search strategy and study selection

The study employed Preferred Reporting Items for Systematic Reviews and Meta- Analysis (PRSMA) guidelines [57]. We performed electronic searches of articles included in this systematic review from the Web of Science, PubMed/Medline and EMBASE from January, 1988 to December, 2019. We combined the following terms: (child or children or childhood or infant or baby or newborn), and (immunization or immunisation or vaccination or vaccine or immunity), and (barrier or hesitant or refuse or refusal or delay or denial or denier or denied or concern or reason or doubt “non-acceptance” or incomplete or obstacle or constraint), and (“Sub Saharan Africa” or Angola or Benin or Botswana or “Burkina Faso” or Burundi or “Cabo Verde” or Cameroon or “Central African Republic” or Chad or Comoros or Congo or “Cote d’Ivoire” or “Equatorial Guinea” or Eritrea or Ethiopia or Gabon or Gambia or Ghana or Guinea or “Guinea- Bissau” or Kenya or Lesotho or Liberia or Madagascar or Malawi or Mali or Mauritania or Mauritius or Mozambique or Namibia or Niger or Nigeria or Rwanda or Senegal or Sierra Leone or Somalia or “South Africa” or “South Sudan” or Sudan or Tanzania or Togo or Uganda or Zambia or Zimbabwe) [See supplementary materials 1].

We restricted to published articles in English that focused on childhood immunization barriers, conducted in Sub-Saharan Africa from January 1988 to December 2019. Articles were excluded if: (i) focused on barriers to immunization for children in other regions of the world, (ii) studied adult immunization barriers (iii) published before December, 1988 and beyond December, 2019; (iv) they were editorials, (v) reports, (vi) review articles, (vii) supplement articles, (vii) bulletins and (ix) studies not available on the university library. We included only observational studies in this systematic review.

### Data analysis

Search result items were uploaded into EndNote X7 library. Duplicates were removed. JBB and DQ did the initial screening (title and abstract) and full texts of articles based on the inclusion and exclusion criteria. We resolved disagreements with third review (FO). The study employed narrative synthesis. The author uses the following approaches: tabulation and thematic analysis. The analysis focus on thematically grouping the barriers identified in the included studies. Researchers formulated table to capture descriptive information and data for each include study. This includes author, year, geographical location and number of countries included in a study; participants and demographic; study design; reported national immunization coverage, data source, date; study quality; and key reported barriers. JBB synthesized data and created table with input from LC and SX. JBB and LC classified reported barriers into three major categories: barriers confronting the parents/caretakers, those specific to the health system, and those linked to the providers. Discrepancies were resolved by consensus after discussions.

### Study methodological quality

**Fig. 1:**
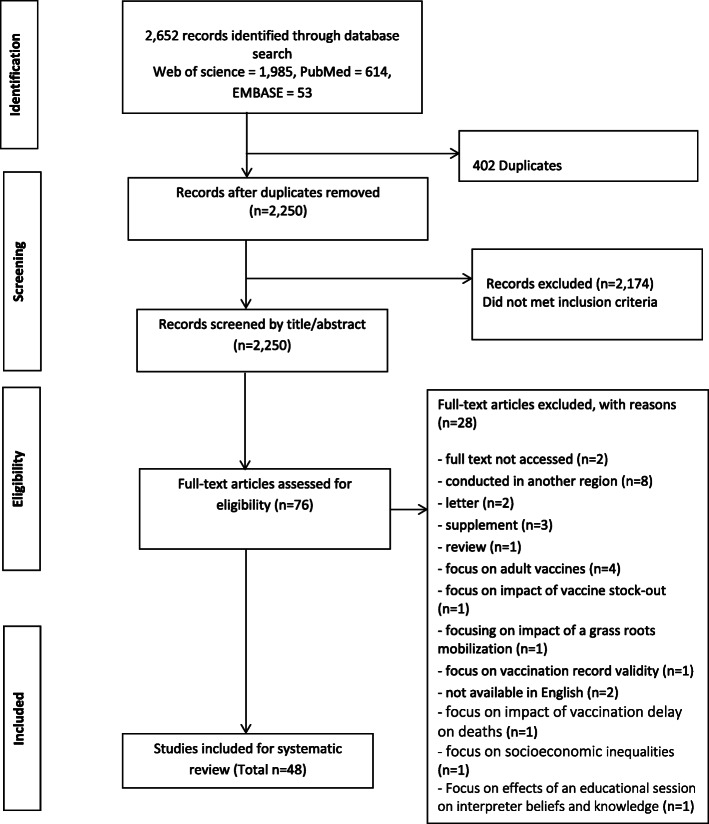
Study selection

JBB and DQ assessed articles for methodological quality independently based on modified tool designed to assess quantitative and qualitative studies used in a similar study published elsewhere [58]. [See supplementary materials 2] It included a range of items from 1 to 14. Each item scores one point. Based on the scores, we grouped articles into three: low, moderate and high; articles scored 12 points and above were considered high methodological quality, moderate 8 to11 points,7 points and below were low. If ratings differed, we discussed the article in an effort to arrive at a consensus (Fig. 1).

## Results

Our database searches yielded 2652 records. 2250 records were screened by title and abstract after duplicates removed; 76 full-text articles assessed for eligibility. Forty-eight articles met all inclusion criteria for this study; 28 articles were excluded for various reasons. [See supplementary materials 3] Nine articles employed second-hand data analysis [7, 12, 18, 27, 37, 40–42, 47]; thirty-six used cross-sectional design [1–3, 11, 13–17, 19, 21–26, 28–36, 38, 39, 42, 45, 46, 48–51, 53, 54] and three used case-control study [20, 43, 44]. All described studies were conducted on Africa populations (103,655 adults and 76,327 children). Forty-seven articles focus on identifying barriers to general childhood vaccination 0–59 months [1–3, 7, 11–17, 19–54] in the following countries: Uganda, Ethiopia, Kenya, Sudan, Nigeria, Gabon, Cameroon, South Africa, Tanzania, Burkina Faso, Togo, Ghana, Malawi and few unspecified countries in Sub-Saharan. One critically examined barriers specific to vaccination doses at birth 0 – 1 day [18] in the Gambia. Thirty articles reported national immunization coverage [1, 2, 7, 11–16, 20–25, 28, 29, 32, 34, 35, 38–40, 43–46, 49, 50, 52–54], eighteen did not [3, 17–19, 22, 24, 26, 27, 30, 31, 33, 36, 37, 41, 42, 47, 48, 51]. 39 articles were classed as high and 9 were moderate methodological quality. We split results into three sections – parental barriers, health system barriers, and providers’ barriers. 8 studies examined all – parental/caretakers, health systems and providers’ barriers [3, 11, 13, 16, 43, 45, 48, 52]; 21 studies examined parental/caretakers and health system barriers [1, 2, 14, 15, 18, 19, 22, 23, 25, 29, 31–33, 35, 38, 44, 47, 49–51, 53]; 18 studies only looked at parental//caretakers barriers [7, 12, 17, 20, 21, 24, 26–28, 34, 36, 37, 39, 42, 46, 54] and one study examined parental and providers’ barriers. Several Sub-Saharan countries were reported to have low childhood immunization coverage with variations across the region. Nigeria reported lowest with 12·7% in 2013 and Ethiopia highest with 88% in 2013. See summary table for studies’ characteristics and key findings (Table 1).

**Table 1:** Summary of studies’ characteristics and key findings

Author, year, geographical location and number of countries included in the study	Participants and demographic	Study design	Reported national immunization coverage, Data source, Date	Quality	Key childhood immunization reported barriers		
					Parental/caretakers barriers	Health systems barriers	Providers barriers
Tadesse et al. [1], 2009 Ethiopia East Africa, 1	126 adults	Cross-sectional	38·5% National health survey of Ethiopia, 2006	Moderate	Misunderstanding of side effects, busy with seasonal farm work	Absence of electricity to maintain the cold chain	
Obasoha et al. [2], 2018 Nigeria West Africa, 1	215 mothers	Cross-sectional	25%, World Health Organization, Global Immunization Vision and Strategies, 2013	High	Unaware of the need of immunization, lack of information, fear of side effect	Vaccines not available, vaccinators absence, long distance to cover	
Malande et al. [3], 2014 Uganda East Africa, 1	311 caretakers/child pairs	Cross -sectional	Not reported	High	Language barrier, less support from husband	Vaccine stock outs, difficult terrain and poor road network, inadequate transportation means, poor working condition of vaccine fridges, inadequate staff, long distance, transportation difficulties	Lack of knowledge of vaccines adverse effects
Wiysonge et al. [7], 2012 Sub-Saharan Africa countries, 24	27,094 children aged 12–23 month	Second-hand data Analysis	71%, WHO (2010); vaccine preventable diseases: monitoring syatem-2010	High	poor households, high illiteracy rates, lack of vaccines information, poor health seeking behaviors (Not going for ANC visit)		
Tefera et al. [11], 2018 Ethiopia East Africa, 1	540 mothers with children aged between 12 and 23 months	Cross -sectional	86%,WHO/UNICEF Immunization coverage,2015	High	fear of side reactions, lack of information, being too busy, place/time being unknown, long waiting time,	vaccinators were absent, vaccines were not available, limited health facilities, vaccine site being too far	
Porth et al. [12], 2019 Ethiopia East Africa, 1	2722 children	Second-hand data Analysis	39%, Ethiopia Demographic and health survey, 2016	High	Negative perception of vaccines, religion, waiting too long	limited operating hours, clinic distance	
Kiptoo et al. [13], 2015 Kenya East Africa, 1	298 mothers/guardians	Cross - sectional	86%, Kenya Expanded program on immunization, 2009	High	lack of knowledge, earning less, many siblings	Inadequate health facilities, long distance for out-reach service	
Cockcroft et al. [14], 2014 Nigeria West Africa, 1	2836 children	Cross-sectional	42%, Nigeria Demographic and Health Survey, 2013	High	Misconception about vaccines, fear of side effect, negligence, being busy with other household work	Lack of vaccines	
Nolna et al. [15], 2018 ameroonWest Africa, 1	1134 caretakers	Cross-sectional	82%, WHO-UNICEF estimates of DPT3 coverage, 2017	High	Lack of money, lack of knowledge of vaccines importance, busy with other seasonal work	Shortage of health personnel, inadequate means of transportation	
Zewdie et al. [16], 2016 Ethiopia East Africa, 1	28 mothers	Cross-sectional	88%, Ethiopia National immunization survey, 2013	Moderate	Lack of information, lack of support from male partners, high workload, fear of mistreatment and lack of cooperation from service providers	Poor arrangement and coordination of immunization services, vaccines stock out, lack of viable defaulter tracking system	Inadequate home visit, lack of commitment, poor counseling skills
Babirye et al. [17], 2011 Uganda East Africa, 1	1000 adults	Cross-sectional	Not reported	High	male partner non-supportive, Lack of clothing, lack of money for vaccine related cost, lack of trust in immunization, fear of associated side effects, less education		
Miyahara et al. [18], 2016 Gambia West Africa, 1	50,455 residents including children	Second-hand data Analysis	Not reported	High	living in urban and peri-urban settings, ethnicity, low maternal education, life style	use of multi-dose vails with limited time, long distance	Use of multi-dose vails with limited time
Pertet et al. [19], 2018 Kenya East Africa, 1	515 mothers	Cross-sectional	Not reported	High	Movement of the whole family (migration)	Lack of vaccines, difficult to access the health facility due to bad terrines	
Yenit et al. [20], 2018 Ethiopia East Africa, 1	308 mothers	Case-control study	39%, Ethiopian Demographic and Health Survey, 2016	High	Delivery at home, lack of antenatal and postnatal care visit, miss conception about vaccines,		
Tugumisirize et al. [21], 2002 Uganda East Africa, 1	408 caretakers	Cross-sectional	29%, Uganda Demographic and Health Survey, 1995	High	Fear of rude health workers, being busy, low level of formal education, fear of side effects, perceived contradictions,	Long distance	
Babalola S [22]., 2011 Nigeria West Africa, 1	882 women	Cross-sectional	Not reported	High	Lack of knowledge about immunization schedule and sources, spouses disapproval of immunization, myth and rumors about side effects, mothers too busy, religious and cultural beliefs, home delivery	Vaccines unavailability, Long distance	
Oladokun et al. [23], 2010 Nigeria West Africa, 1	248 mothers	Cross-sectional	12·7% National immunization survey, 2003	High	Religion, low mother’s education, mothers not being aware of additional doses	Non-availability of vaccines,	
Schwarz et al. [24], 2009 Gabon West Africa, 1	262 mothers	Cross-sectional	Not reported	Moderate	feeling ashamed of poverty-associated reasons such as poorly cloth child or dirty, lack of knowledge,	Transportation cost, long distance	
Ismail et al. [25], 2014 Sudan East Africa, 1	213 children	Cross-sectional	60%, Federal Ministry of Health, 2005	High	lack of knowledge,, mothers too busy, many siblings, fear of side effect	Vaccinators absence, vaccine stock out, long distance	
Rees et al. [26], 1991 South Africa South Africa, 1	315 women	Cross-sectional	Not reported	High	low literacy level of mothers	Long distance to reach facility	
Nadella et al. [27], 2019 Tanzania East Africa, 1	31,999 children	Secondary data analysis	Not reported	High	Parents not educated, mothers not attending ANC, delivery at home, poor household		
Meleko et al. [28], 2017 Ethiopia East Africa, 1	322 mothers/caretakers	cross-sectional	24.3% Ethiopia Demographic Health Survey (EDHS), 2011	High	Low parental educational level, delivery at home, parents not utilizing maternal health care services. Lack of knowledge		
Itimi et al. [29], 2012 Nigeria west Africa, 1	558 women	cross-sectional	23% Nigeria Demographic and Health Survey, 2008	High	Adverse rumor about childhood immunization	Inadequate health personnel (Vaccinators)	
Kagoné et al. [30], 2018 Burkina Faso West Africa, 1	Not stated	cross-sectional	Not reported	Moderate	Migration, mothers being busy, poor interaction between women and health workers, potential adverse events, and lack of information.	Geographic (hard to reach arears)	Not open multi-dose vails unless a critical number of children are present
Tobin-West et al. [31], 2012 Nigeria West Africa, 1	1560 mothers/caregivers	cross-sectional	Not reported	High	Long waiting time, belief in the efficacy of traditional medicines as an alternative to immunization, poor rapport with health workers,	Frequent shortage of vaccine	
Braka et al. [32], 2011 Uganda East Africa, 1	136 caretakers	cross-sectional	80% W H O Immunization profile—Uganda 1980–2008, 2010	High	Misconceptions, adverse effects experience, providers’ bad attitudes	Inadequate staff at health center level, poor storage facilities for vaccines,	
Ambe et al. [33], 2001 Nigeria West Africa, 1	500 mothers	cross-sectional	Not reported	High	Parents don’t have trust in vaccines, parents are abused in hospitals, husband refused/not supportive	Vaccines not available	
Tadesse et al. [34], 2009 Ethiopia East Africa, 1	226 children	cross-sectional	49.9% Federal Ministry of Health Ethiopia, 2006	High	Poor knowledge about immunization, mother’s negative perceptions, low monthly income of parents		
Jani et al. [35], 2008 Mozambique South Africa, 1	668 mothers	cross-sectional	80% Expanded program on immunization, 1998	High	Low education level of mothers, long waiting time for vaccination, parent’s forgetfulness, migration, concomitant treatment by traditional healers	Vaccines shortage, Inadequate health workers (Vaccinators)	
Eng et al. [36], 1991 Togo West Africa, 1	110 mothers/caretakers	cross-sectional	Not reported	Moderate	Lack of knowledge, parent’s forgetfulness, health workers being aggressive, long waiting time, laziness, lack of information about vaccines, low income of parents	Long distance to health facilities	
Landoh et al. [37], 2016 Togo West Africa, 1	2067 children (12 to 59 months)	Secondary data analysis	Not reported	High	Residence of mother (Muslims dominated), non-schooled mothers, being a single mother, negative cultural beliefs		
Legesse et al. [38], 2015 Ethiopia East Africa, 1	591 children 12 to 23 months and their mothers	cross-sectional	36.5% Ethiopia Demographic Health Survey (EDHS), 2011	High	Lack of knowledge, lack of information, low family income, low education level of parents, low maternal health care utilization, fear of adverse reactions, lack of trust on immunization, male partners non supportive	Poor quality of health information regarding immunization, Long distance to health facilities	
Wemakor et al. [39], 2018 Ghana West Africa, 1	322 children and their mothers	cross-sectional	77% Ghana Demographic and Health Survey 2014		Community of residence of mothers, lack of knowledge,		
Adedokun1et al [40], 2017 Nigeria West Africa, 1	5754 children aged 12–23 months	Secondary data analysis	81.5% Federal Ministry of Health Nigeria, 2011	Moderate	Mothers being illiterate, lack of information about immunization, mothers not attending ANC, delivery at home, economically dis advantage mothers, difficulty getting to health facility due to bad terrines		
Chidiebere et al. [41], 2014 Nigeria West Africa, 1	34,596 women	Secondary data analysis	Not reported	High	Lack of information about immunization, fear of side-effects, delivery at home, place of residence	Immunization centers too far	
Ekouevi et al. [42], 2018 Togo West Africa, 1	1128 children aged 12–23 months	cross-sectional	Not reported	High	Mothers not educated, low income, poor road conditions,,	Lack of means of transportation, Long distance to health center	
Tadess et al. [43], 2017 Ethiopia East Africa, 1	630 mothers/caretakers	Case control	79% Ethiopian Health Sector Transformation Plan (HSTP), 2009	High	Inaccessible health facility, poor motivation, unfavorable attitude and bad treatment of health workers, lack of logistics, inconvenient immunization time, inadequate information about immunization	Vaccines shortages	Inadequate communication skills, lack of willingness and restricted vaccine open policy,
Negussie et al. [44], 2016 Ethiopia East Africa, 1	548 children aged 12 to 23 months	Case control	24% Ethiopia Demographic and Health Survey, 2011	High	Lack of knowledge about immunization benefits, mother’s negative perception of vaccine side effects, migration of mothers	Unavailability of vaccines	
Bosu et al. [45], 1997 Ghana West Africa, 1	469 mothers	Cross sectional	43% Ministry of Health Ghana Maternal and Child Health and Family Planning. Annual Report, 1992	High	Poor knowledge about immunization, financial difficulties, long waiting times,, attitude of service providers and fear of side-effects	Lack of suitable venues and furniture at outreach clinics, and weak inter-sectoral collaboration, transport difficulties	Poorly motivated service providers
Desgrées du Loû et al. [46], 1994 Senegal West Africa, 1	6078 Mothers/caretakers	Cross sectional	51% WHO/EPI/CEIS/93.1 (summary for the WHO African Region) 1990	Moderate	children in large compound with large number of children.	Distance between the child and the health center, difficult geographical terrain	
Sato R [47]., 2019 Nigeria West Africa, 1	28,085 children	Secondary data analysis	Not reported	Moderate	Have no faith in immunization, lack of awareness of the need for immunization, poor household	Shortage of vaccine, limited health centers immunization point is too far/inconvenient	
Akwataghibe, N. N. et al. [48], 2019 Nigeria West Africa, 1	215 children,	Cross sectional	Not reported	High	Ethnicity, culture, household decision making, and gender relations; lack of knowledge and awareness of the value of immunization, negative beliefs and attitudes toward immunization; past experiences with immunization, migration	shortage of health workers, unavailability of vaccines at scheduled times; Inadequate electrical power supply to keep the vaccine cold chain at facilities, long distances for mothers in hard-to-reach areas	Reminders not sent on time about routine immunization or outreach days
Yismaw, A. E. et al. [49], 2019 Ethiopia East Africa, 1	301 mothers/caretakers	cross-sectional	86% Federal Ministry of Health (2010)	High	Lack of Knowledge of next visit; and lack of knowledge about the benefits of vaccination	long distances to reach nearby health facilities	
Ntenda P [50]., 2019 Malawi East Africa, 1	3111 children and mothers	Cross-sectional	76% WHO (2015)	High	Children born to mothers without education, children poor households, mothers with many sibling, children whose delivery occurred at home,	Inadequate health facility for vaccination, long distances to reach nearby health facilities	
Okenwa, U. J. et al. [51], 2019 Nigeria West Africa, 1	344 mothers and their infant	Cross-sectional	Not reported	High	Lack of awareness on timing of valid vaccine,	Vaccine stock-out at the immunization site	
Mthiyane, T. N et al. [52], 2019 South Africa South Africa 1	847 eligible children aged 12–59 months	Secondary data analysis	66% WHO/UNICEF (2015)	High	Low household monthly income, unfriendly health workers,	Vaccine shortage; long distance and transportation costs to reach the clinic for immunization	Low level of education of the primary caregiver,
Mekonnen, A. G. et al. [53], 2019 Ethiopia East Africa, 1	566 children aged 12–23 months and their mothers/caregivers	Cross sectional	39% Ethiopian demographic health survey report (2016)	High	Forgotten appointment date, the experience of child sickness with previous vaccination, and disrespectful behavior of health professionals	Long distance to the clinic	
Ibraheem, R. et al. [54], 2019 Nigeria West Africa, 1	480 mother-infant	cross-sectional	53% Nigeria immunization coverage survey (2016)	High	Lack of antenatal care visit, vaccination on weekend/public holidays lower educational level		

### Parental/caretaker barriers

In this systematic review, several cited parental/caretaker’ barriers were modifiable (knowledge, misconception, trust, delivery at home, long waiting time, providers’ hostility, parent’s forgetfulness, inconvenient time and language barrier). Parental/caretaker barriers are factors that impede mothers/caretakers progress in the process of their child accessing and utilizing vaccine services. It was revealed that parent perception influenced immunization of their children [1, 12–14, 21, 34–44, 48, 49]. Parents not being knowledgeable of immunization was the most frequently and consistently reported barrier to childhood immunization [2–40, 42, 44, 45, 47–50, 52]. Wiysonge et al. (2012) stated that “low parental knowledge of immunization and/or lack of access to information about childhood immunization could be an important contributor to the high burden of unimmunized children in sub-Saharan Africa”. Four studies [3, 13, 25, 26], noted that a child born to a mother with little or no knowledge of vaccination may not complete the required vaccine series. Two articles reported that delay on vaccine birth doses is associated with maternal education [18, 20]. Misconceptions about childhood immunization were recorded as major hindrance to effective utilization of immunization services in this review [1, 2, 11–14, 21, 22, 29, 31, 32, 48, 49] One article [17] reported that some parents believed that the immunity induced by vaccines is less effective than that of the natural disease, and they prefer to endure the diseases than immunization. Some caregivers were reported to believe in the efficacy of traditional medicines as an alternative to immunization and concomitant treatment by traditional healers [31, 35].

Lack of trust towards vaccines was a major reported barrier. Some community members were reported to refuse immunization services due to the belief that vaccines were ‘harmful’, ‘expired’ and could cause ‘physical disability’ and/or ‘death’ among their children [2, 17, 21, 22, 30, 32, 33, 35, 38, 45, 48, 53]. The place of delivery of a baby was reported as determinants of full immunization of a child. Delivery at health facility enhances full immunization [18, 20, 22, 27, 28, 40, 48]. Long waiting time at health facilities was frequently and consistency noted [11, 12, 31, 35, 36, 45]. Two articles [35, 53], noted that parents sometimes forgot the appointment date for the next immunization visit of their children. Others reported place/time for vaccination being unknown [11]. Inconvenient immunization time such as on weekend/public holidays was reported as a barrier [43, 54]. One study [3], indicated language as a barrier to childhood immunization.

On the other hand, we also recorded non-modifiable childhood immunization barriers of parents/caretakers. Those categorized as unmodifiable are factors that are extrinsic to the parent / provider dyad. These include occupation, financial limitations, place of residence of mother/caretaker, religion, ethnicity, family size, male partners’ support, and migration; seasonal farm work, feeling ashamed of poverty-associated reasons, and being a single mother. The role of male partners in the decision for childhood vaccination was an important barrier noted. Male partners were often cited as being against vaccinating the children. The decision for immunization was generally a joint decision between the mother and father of the child. But it was noted with strong emphasis that women were in charge of taking children for immunization and sometimes the husbands opposed immunization and stopped their wives from immunizing their children by denying them the social and financial support necessary [3, 16, 17, 22, 33, 38]. The nature of occupation of the mother/caretaker was reported as a major determinant to childhood immunization [1, 11, 14, 15, 21, 22, 25, 30]. Housewives were reported to have complied with higher coverage of full immunization status than other occupations such as merchants or public/private employees [11]. Also, mothers/caretakers were reported to be affected by seasonal factors. One study [1] stated that, “usually in the first quarter of the year in which most mothers engaged in coffee-collection and processing in coffee processing industries often did not bring their children to the next immunization schedule”. Financial limitation was a major barrier cited that hinder childhood immunization [7, 11, 13, 15, 17, 24, 27, 34, 36, 38, 40, 42, 45, 47, 50, 52]. The place of residence of the mother was reported as determinants of full immunization of a child [37, 39, 41]. One study noted that the likelihood of vaccination of a child by day 7 is higher among children residing in rural areas than those in urban and pre-urban settings [18]. Socio-cultural factors and religion were noted to have negatively impacted immunization uptake [12, 18, 19, 22, 23, 37, 48]. Ethnicity and cultural beliefs were reported barriers to vaccine utilization and coverage; certain ethnic groups within the same country were identified with low coverage. Family size was associated with the probability of a child being fully immunized. It was revealed that children from large families have low vaccine uptakes, considering the burden of other children at home in taking up immunization services [11, 13, 25, 46, 50]. Migration was also cited as a hindrance to childhood immunization coverage [30, 35, 44, 48]. Feeling ashamed of poverty-associated reasons was reported as barrier. Schwarz et al. (2009) indicated that “mothers who felt that they could not dress smartly enough for the approval of other women at the clinic were less likely to attend” [24]. Babirye et al. (2011) further revealed that “poor mothers often felt stigmatized and bullied from other women and health workers if they did not show up in good clothing” [17] Being a single mother was also a cited barrier to childhood immunization in this review [37].

### Health system barriers

We noted health system barriers in this review. We describe health system barriers as inherent factors that obstruct the process of delivering vaccine and vaccine related services to it beneficiaries. These includes broken cold chain, irregular supplies and distribution of vaccines; limited human resource and infrastructures, and long distances separating health facilities from families [1–3, 11, 13–16, 19, 22, 23, 25, 29, 32, 35, 43, 44, 47–52]. Vaccine shortages at health facility level and difficulties of transporting vaccines were commonly reported to significantly hinder immunization services [1–174 3,11,14,16,19,22,23,25,33,43,47,48,52,51]. Some facilities were reported to have utilized vaccine refrigerators from nearby health centers due to poor working condition of theirs [1, 3, 32]. It was noted that due to staff limitation, only one staff often conducted immunization sessions in the catchment population [2, 3, 11, 25]. Studies [11, 13, 45, 47–50, 52, 53] also revealed that some hard-to-reach areas do not have health facilities nearby to provide childhood immunization. Health workers were reported to covers long distances on outreach services due to inadequate health centers [11, 13, 15, 43]. It was also reported that, caretakers covered long distances to reach immunization centers resulted to non-completion of vaccination series [2, 3, 13, 18, 21–26, 31, 36, 38, 40–43, 46]. Some studies [11, 13, 18, 26] attempted to analyze the associations of distance with immunization outcomes. Tefera et al. (2018) indicated that “families whose home was at least an hour from the vaccination site were less likely to be fully vaccinated (56%) than families whose home was between 30 and 59 min away (67%)”. According to Miyahara et al. 2016, “the longer the distance from vaccination site, the lower the chances of vaccination by day 7 (of life) of a child”. Poor arrangement and coordination of immunization seasons at health center level were identified as barrier [16, 38, 43, 45].

### Providers barriers

In addition to the parental and health system barriers mentioned above, providers were identified as possessing barriers to immunization. Providers’ barriers are those factors that limit the process of health service providers to fully provide vaccine services to it beneficiaries. These factors include the lack of knowledge of vaccine indications and contraindications and the lack of counseling skills [3, 16, 43, 52]. The restricted vaccine opening policy (use of multi-dose vials and the limited time for their use) was noted as a barrier specifically for the BCG vaccine [18, 30, 43]. It was also cited that reminders were not sent on time about routine immunization or outreach days [48] Providers’ hostility and rude attitudes to mothers were also a reported immunization barriers in this review [15, 16, 24, 30–33, 36, 45, 52, 53].

## Discussion

Our review aims at identifying major childhood immunization barriers confronting health systems, providers, and parents across sub-Saharan Africa. Understanding of these barriers will help inform decision-makers and other relevant players involved in immunization programs, and to guide health interventions aim at improving immunization coverage. The study revealed childhood immunization barriers affecting utilization and coverage in the region. We grouped these barriers under three separate domains: barriers inherent in the parents/caretakers, those specific to the health system, and those related to the providers. We acknowledge that the categorisation of barriers may be different in this review than in others. Parental barriers were more and consistently identified than providers and health systems. Several of the cited parental/caretakers’ barriers were unmodifiable. Parents/caretakers reported barriers include lack of knowledge, misconceptions, financial deprivation, lack of partners’ support, and distrust of the medical systems. Other associated factors include the number of offspring, life style, migration, place of residence, long waiting time, parent’s forgetfulness; inconvenient time, being a single mother, occupation, language barrier, seasonal farm work, and feeling ashamed of poverty-associated reasons. Health system barriers include inadequate infrastructures and cold chain maintenance; distance and poor coordination. Providers’ constraints include limited human resources, hostile attitude and knowledge.

Knowledge of vaccines is very important for effective vaccine acceptance and utilization by parents. Low vaccination coverage in children is largely a result on the lack of knowledge of vaccines of healthcare providers and parents. Parents with low education and low socioeconomic status attainment showed more uncertainty towards immunization [3, 11, 13, 15–18, 22, 24, 25, 27, 28, 30, 34–36, 40, 42, 44, 45, 48, 49]. This result was also mirrored in another systematic review conducted in middle and low income countries which revealed that, most often, strong tie exist between low socioeconomic status and low level educational; with potential to lower vaccine coverage. However, investigations to understand the dynamics of these relationships are not sufficient [55]. Thus health education programs targeting these groups are critical in increasing vaccines acceptance, utilization and coverage. Further studies to unearth the dynamics of these relationships are vital. We noted that parents held reservations towards the associated side effects of vaccines. Other expressed a total distrust of immunization programs and vaccines [2, 17, 21, 22, 30, 32, 33, 35, 38, 45, 47, 48]. This is in line with previous review of Influenza Vaccine hesitancy, which pointed out that, a lack of confidence due to low perceived effectiveness of the vaccine was a hindrance to vaccine uptake [56]. Another review outlined similar beliefs, including concerns about side effects, uncertainty toward vaccine safety, and belief in anti-vaccine theories [59]. To overcome this, immunization programs should intensify public sensitization on vaccines safety and promote effective mechanisms of addressing parents’ concerns. Healthcare workers should develop approaches that acknowledge parental concerns and respectfully try to correct their misconceptions. The attitude of male partners against immunization is often noted in this review. A study carried out in Cambodia suggested that women’s decision-making power and autonomy were relevant to maternal and child health outcomes [60] It is important to carefully consider the social contexts during program design and implementation for child immunization. We need to effectively address socio-cultural contexts by involving the entire community, and not only target mothers and female caregivers. The review also raised the pressing need for women to be empowered to overcome their financial challenges in taking their children to vaccination centers.

Equally challenging is overcoming health system barriers identified, including staff shortage, the cost of maintaining the cold chain, storage and transportation of vaccines and consumables. The long distances between health centers and the families they serve are barrier that require systemic policy changes to address. The data suggest that countries should increase government financial gross domestic product (GDP) allocation to their health sector, consistent with the recommendation in the Abuja declaration [61]. Increased financial resources would enable countries to equip and upgrade existing health facilities and to increase their numbers. Targeted resources may motivate and enable staff deployed in remote areas for effective outreach activities to maximize coverage of immunization. Poor arrangement and coordination of immunization seasons at health-center level was noted [16, 38, 43, 45] this findings reflect a review (conducted in sub-Saharan countries) focus on children and youth which noted that poorly organized services can cause delays and increase costs for beneficiaries [62]. A coordinated National Immunization Program can rationalize services, thus improve immunization uptake and regulating healthcare providers.

In this review of barriers to childhood immunization, the parental/caretaker’ barriers were mostly identified, followed by health systems and providers’ barriers. It corroborates a systematic review (studies undertaken across countries) which noted that, family characteristics, parents’ understanding about vaccines and attitude were marked factors to non-immunization of their children. These pose a challenge to immunization programs due to its complexity and require strategic interventions [63]. A published article exploring vaccine hesitancy stated that various attitudes seems to result into specific categories; for instance, vaccine refusal attitude could be as a result of having little or no knowledge about vaccine, lack of trust on the vaccine or it could as well linked to financial limitations [64]. This finding disagreed with previous systematic review conducted in middle- and low -income countries. It indicated that the main factors that impede vaccination uptake and coverage were associated with healthcare system [55]. Some of the barriers cited may be modifiable within the constraints of overstretched health systems. Others may require systemic policy changes to address. Some healthcare system related factors can be realistic to design strategies that can be implemented in a range of settings, such as training of health workers to reduce missed opportunities, improve communication, and remove barriers by enhancing outreach services.

### Study limitations

Our study acknowledged and outlined few limitations. As most literatures cited are observational in nature, this study cannot confirm causation nor completely rule out confounding. A few studies also relied on survey data [7, 12, 18, 27, 37, 40, 41] with the potential for selection or nonresponse bias. Population-based data studies may be liable to misclassification or measurement error, leading to information biases. Retrospective studies of caretakers/parents beliefs are subject to recall bias. Lack of protocol registration of this review may limit the evidence to demonstrate that, components of the research plan have been fully addressed. The review also lack grey literature/unpublished literature searching with potential for publication bias. Majority of the studies were conducted in East Africa [1, 3, 11–13, 16, 17, 19–21, 25, 27, 28, 32, 34, 38, 43, 44] and West Africa [1, 15, 18, 22–24, 29–31, 33, 36, 37, 39–42, 45, 46] limiting generalizability to the rest of the continent. A quantitative meta-analysis from these studies may have been useful for analyzing quantitative trends, although the heterogeneity of the studies precluded such analyses.

## Conclusion

Although various methods of improving vaccination coverage in sub-Saharan Africa have been identified, achieving the desired levels for the realization of the fullest benefits of immunization is still a major challenge. This can be achieved through combined efforts of healthcare systems and providers; and address people, the communities and societies in which they live. Aggregation of known immunization barriers and the evidence on effective interventions to address these barriers should be core component of immunization programs in Sub-Saharan Africa and elsewhere.

## Supplementary information

**Additional file 1.** Database search terms and history. Methodological Quality Assessment tool for Qualitative and Quantitative studies. Full-text articles excluded with reasons.

## Data Availability

Not applicable.

## Abbreviations

DTP3: Diphtheria–tetanus-pertussis vaccine third dose
BCG: Bacillus calmette–guérin
PRSMA: Preferred reporting items for systematic reviews and meta- analysis
GDP: Gross domestic product
