# Supplementary material for: Barriers to childhood immunization in sub-Saharan Africa: A systematic review
Source: BMC Public Health. 2020 Jul 14;20:1108. doi: 10.1186/s12889-020-09169-4 (PMC7362649; doi:10.1186/s12889-020-09169-4)
Supplement: Supplementary file 1 — Additional file 1. Database search terms and history. Methodological Quality Assessment tool for Qualitative and Quantitative studies. Full-text articles excluded with reasons. [file 12889_2020_9169_MOESM1_ESM.pdf]

Supplementary Materials

1. **Database search terms and history**
2. **Methodological Quality Assessment tool for Qualitative and Quantitative studies**
3. **Full-text articles excluded with reasons**
4. **Change of authorship request form**

1. Database search terms and history

a) Embase search terms and history:

53 ('child'/exp OR child OR 'children'/exp OR children OR 'childhood'/exp OR childhood OR 'infant'/exp OR infant OR 'baby'/exp OR baby OR 'newborn'/exp OR newborn) AND ('immunization'/exp OR immunization OR 'immunisation'/exp OR immunisation OR 'vaccination'/exp OR vaccination OR 'vaccine'/exp OR vaccine OR 'immunity'/exp OR immunity) AND (('barrier'/exp OR barrier OR hesitant OR 'refuse'/exp OR refuse OR refusal OR delay OR 'denial'/exp OR denial OR denier OR denied OR concern OR reason OR 'doubt'/exp OR doubt) AND 'non acceptance' OR incomplete OR obstacle OR 'constraint'/exp OR constraint) AND ((sub AND saharan AND ('africa'/exp OR africa) OR 'angola'/exp OR angola OR 'benin'/exp OR benin OR 'botswana'/exp OR botswana OR 'burkina faso'/exp OR 'burkina faso' OR 'burundi'/exp OR burundi OR 'cabo verde'/exp OR 'cabo verde' OR 'cameroon'/exp OR cameroon OR 'central african republic'/exp OR 'central african republic' OR 'equatorial guinea'/exp OR 'equatorial guinea' OR 'eritrea'/exp OR eritrea OR 'ethiopia'/exp OR ethiopia OR 'gabon'/exp OR gabon OR 'gambia'/exp OR gambia OR 'ghana'/exp OR ghana OR 'guinea'/exp OR guinea OR 'guinea-bissau'/exp OR 'guinea-bissau' OR 'kenya'/exp OR kenya OR 'lesotho'/exp OR lesotho OR 'liberia'/exp OR liberia OR 'madagascar'/exp OR madagascar OR 'malawi'/exp OR malawi OR 'mali'/exp OR mali OR 'mauritania'/exp OR mauritania OR 'mauritius'/exp OR mauritius OR 'mozambique'/exp OR mozambique OR 'namibia'/exp OR namibia OR 'niger'/exp OR niger OR 'nigeria'/exp OR nigeria OR 'rwanda'/exp OR rwanda OR 'senegal'/exp OR senegal OR sierra) AND leone OR 'somalia'/exp OR somalia OR 'south africa'/exp OR 'south africa' OR 'south sudan'/exp OR 'south sudan' OR 'sudan'/exp OR sudan OR 'tanzania'/exp OR tanzania OR 'togo'/exp OR togo OR 'uganda'/exp OR uganda OR 'zambia'/exp OR zambia OR 'zimbabwe'/exp OR zimbabwe) AND [article]/lim AND [humans]/lim AND ([embase]/lim OR [medline]/lim)

b) Web of Science search terms and history:

1,985 TS=((child or children or childhood or infant or baby or newborn) and (immunization or immunisation or vaccination or vaccine or immunity) and (barrier or hesitant or refuse or refusal or delay or denial or denier or denied or concern or reason or doubt "non acceptance" or incomplete or obstacle or constraint) and (Sub Saharan Africa or Angola or Benin or Botswana or "Burkina Faso" or Burundi or "Cabo Verde" or Cameroon or "Central African Republic" or Chad or Comoros or Congo or "Cote d'Ivoire" or "Equatorial Guinea" or Eritrea or Ethiopia or Gabon or Gambia or Ghana or Guinea or "Guinea-Bissau" or Kenya or Lesotho or Liberia or Madagascar or Malawi or Mali or Mauritania or Mauritius or Mozambique or Namibia or Niger or Nigeria or Rwanda or Senegal or Sierra Leone or Somalia or "South Africa" or "South Sudan" or Sudan or Tanzania or Togo or Uganda or Zambia or Zimbabwe))
Databases= WOS, BCI, KJD, MEDLINE, RSCI, SCIELO Timespan=All years; Search language=Auto

c) PubMed search terms and history

Search	Query	Items found
#14	Search (((#3) AND #6) AND #10) AND #13	614
#13	Search (#11) OR #12	369841

#12	Search (((((((((((((((((((((((((((((((((((((((Sub Saharan Africa[Title/Abstract]) OR Angola[Title/Abstract]) OR Benin[Title/Abstract]) OR Botswana[Title/Abstract]) OR Burkina Faso[Title/Abstract]) OR Burundi[Title/Abstract]) OR Cabo Verde[Title/Abstract]) OR Cameroon[Title/Abstract]) OR Central African Republic[Title/Abstract]) OR Chad[Title/Abstract]) OR Comoros[Title/Abstract]) OR Congo[Title/Abstract]) OR Cote d'Ivoire[Title/Abstract]) OR Equatorial[Title/Abstract]) OR Guinea[Title/Abstract]) OR Eritrea[Title/Abstract]) OR Ethiopia[Title/Abstract]) OR Gabon[Title/Abstract]) OR Gambia[Title/Abstract]) OR Ghana[Title/Abstract]) OR Guinea[Title/Abstract]) OR Guinea-Bissau[Title/Abstract]) OR Kenya[Title/Abstract]) OR Lesotho[Title/Abstract]) OR Liberia[Title/Abstract]) OR Madagascar[Title/Abstract]) OR Malawi[Title/Abstract]) OR Mali[Title/Abstract]) OR Mauritania[Title/Abstract]) OR Mauritius[Title/Abstract]) OR Mozambique[Title/Abstract]) OR Namibia[Title/Abstract]) OR Niger[Title/Abstract]) OR Nigeria[Title/Abstract]) OR Rwanda[Title/Abstract]) OR Senegal[Title/Abstract]) OR Sierra Leone[Title/Abstract]) OR Somalia[Title/Abstract]) OR South Africa[Title/Abstract]) OR South Sudan[Title/Abstract]	284064
#11	Search Sub Saharan Africa[MeSH Terms]	192421
#10	Search (#7) OR #9	778722
#9	Search (((((((((((((barrier[Title/Abstract]) OR barrier[Title/Abstract]) OR hesitan*[Title/Abstract]) OR refus*[Title/Abstract]) OR delay[Title/Abstract]) OR denial[Title/Abstract]) OR denier[Title/Abstract]) OR denied[Title/Abstract]) OR concern[Title/Abstract]) OR reason[Title/Abstract]) OR doubt[Title/Abstract]) OR non acceptance[Title/Abstract]) OR incomplete[Title/Abstract]) OR obstacle[Title/Abstract]) OR constraint[Title/Abstract]	778722
#8	Search barrier[MeSH Terms] Schema: all	0
#7	Search barrier[MeSH Terms]	0
#6	Search (#4) OR #5	948974
#5	Search (((((immunization[Title/Abstract]) OR immun*[Title/Abstract]) OR vaccination[Title/Abstract]) OR vaccine[Title/Abstract]) OR immunity[Title/Abstract]) OR vaccine*[Title/Abstract]	902488
#4	Search immunization[MeSH Terms]	166811
#3	Search (#1) OR #2	2465532
#2	Search (((((child[Title/Abstract]) OR children[Title/Abstract]) OR childhood[Title/Abstract]) OR infant[Title/Abstract]) OR baby[Title/Abstract]) OR newborn[Title/Abstract]	1527863
#1	Search child[MeSH Terms]	1820272

2. Modified Methodological Quality Assessment tool for Qualitative and Quantitative studies

Appraisal questions:

1. Did the study address a clearly focused question / issue?
2. Is the research method (study design) appropriate for answering the research question?
3. Is the method of selection of the subjects (employees, teams, divisions, organizations) clearly described?
4. Could the way the sample was obtained introduce (selection) bias?
5. Was the sample of subjects' representative with regard to the population to which the findings will be referred?
6. Were the data collected in a way that addressed the research issue?
7. Was the sample size based on pre-study considerations of statistical power?
8. Was a satisfactory response rate achieved?
9. Are the measurements (questionnaires) likely to be valid and reliable?
10. Have ethical issues been taken into consideration?
11. Was the data analysis sufficiently rigorous?
12. Was the statistical significance assessed?
13. Is there a clear statement of findings?
14. Can the results be applied to your organization or field of study?

Key: NA: Not Applicable; NC: Not Clear; N: No; Y: Yes

Modified Methodological Quality Assessment tool for Qualitative and Quantitative studies															
**Article, year**	**Appraisal questions**													**Remarks**	
	1	2	3	4	5	6	7	8	9	10	11	12	13	14	Total scores	Quality status
Tadesse et al [1], 2009	Y	Y	Y	N	Y	Y	NA	Y	NC	N	Y	NA	Y	Y	10	Moderate
Phillips et al [2], 2018	Y	Y	Y	N	Y	Y	Y	Y	Y	Y	Y	Y	Y	Y	14	High
Oliver et al [3], 2014	Y	Y	Y	N	Y	Y	Y	Y	Y	Y	Y	Y	Y	Y	14	High
Wiysonge et al [7], 2012	Y	Y	Y	N	Y	Y	Y	Y	Y	Y	Y	Y	Y	Y	14	High
Yemesrach et al [11], 2018	Y	Y	Y	N	Y	Y	Y	N	Y	Y	Y	Y	Y	Y	14	High
Porth et al [12], 2019	Y	Y	Y	N	Y	Y	Y	Y	Y	Y	Y	Y	Y	Y	13	High
Elizabeth et al [13], 2015	Y	Y	Y	N	Y	Y	Y	Y	Y	Y	Y	Y	Y	Y	14	High
Cockcroft et al [14], 2014	Y	Y	Y	N	Y	Y	Y	Y	Y	Y	Y	Y	Y	Y	14	High
Sylvie et al [15], 2018	Y	Y	Y	N	Y	Y	Y	Y	Y	Y	Y	Y	Y	Y	14	High
Zewdie et al [16], 2016	Y	Y	Y	N	Y	Y	NA	Y	NC	Y	Y	NA	Y	Y	11	Moderate

Juliet et al,[17] 2011	Y	Y	Y	N	Y	Y	NA	Y	Y	Y	Y	NA	Y	Y	12	High
Miyahara et al [18], 2016	Y	Y	Y	N	Y	Y	Y	Y	NC	Y	Y	Y	Y	Y	13	High
Pertet et al [19], 2018	Y	Y	Y	N	Y	Y	Y	Y	Y	Y	Y	Y	Y	Y	14	High
Yenit et al [20], 2018	Y	Y	Y	N	Y	Y	Y	Y	Y	Y	Y	Y	Y	Y	14	High
Tugumisirize et al [21], 2002	Y	Y	Y	N	Y	Y	N	Y	Y	Y	Y	Y	Y	Y	13	High
Babalola S [22], 2011	y	Y	Y	N	Y	Y	Y	Y	Y	Y	Y	Y	Y	Y	14	High
Oladokun et al [23], 2010	Y	Y	Y	N	Y	Y	Y	Y	Y	Y	Y	Y	Y	Y	14	High
Schwarz et al [24], 2009	Y	Y	Y	Y	Y	Y	N	Y	NC	Y	Y	NA	Y	Y	10	Moderate
Ismail et al [25], 2014	Y	Y	Y	N	Y	Y	Y	Y	Y	Y	Y	Y	Y	Y	14	High
Rees et al [26], 1991	y	Y	Y	N	Y	Y	Y	Y	Y	Y	Y	N	Y	Y	13	High
Nadella et al [27], 2019	Y	Y	Y	N	Y	Y	Y	Y	Y	Y		Y	Y	Y	14	High
Meleko et al [28], 2017	Y	Y	Y	N	Y	Y	Y	Y	Y	Y	Y	Y	Y	Y	14	High
Itimi et al [29], 2012	Y	Y	Y	N	Y	Y	Y	Y	Y	Y	Y	Y	Y	Y	14	High
Kagoné et al [30], 2018	Y	Y	Y	NC	Y	Y	NA	Y	Y	Y	Y	NA	Y	Y	11	Moderate
Tobin-West et al [31], 2012	Y	Y	Y	N	Y	Y	Y	Y	Y	Y	Y	Y	Y	Y	14	High
Braka et al [32], 2011	Y	Y	Y	N	Y	Y	N	Y	Y	Y	Y	NA	Y	Y	12	High
Ambe et al [33], 2001	Y	Y	Y	N	Y	Y	N	Y	Y	Y	Y	Y	Y	Y	12	High
Tadesse et al [34], 2009	Y	Y	Y	N	Y	Y	Y	Y	Y	Y	Y	Y	Y	Y	14	High
Jani et al [35], 2008	Y	Y	Y	N	Y	Y	N	Y	Y	Y	Y	Y	Y	Y	13	High

Eng et al [36], 1991	Y	Y	Y	NC	Y	Y	N	Y	Y	Y	Y	N	Y	Y	11	Moderate
Landoh et al [37], 2016	Y	Y	Y	N	Y	Y	N	Y	Y	Y	Y	Y	Y	Y	14	High
Legesse et al [38], 2015	Y	Y	Y	N	Y	Y	Y	Y	Y	Y	Y	Y	Y	Y	14	High
Wemakor et al [39], 2018	Y	Y	Y	N	Y	Y	Y	Y	Y	Y	Y	Y	Y	Y	14	High
Adedokun1et al [40], 2017	Y	Y	Y	N	Y	Y	NC	NC	NA	NA	Y	Y	Y	Y	10	Moderate
Chidiebere et al [41], 2014	Y	Y	Y	N	Y	Y	NC	NC	NA	NA	Y	Y	Y	Y	10	Moderate
Ekouevi et al [42], 2018	Y	Y	Y	N	Y	Y	Y	Y	Y	Y	Y	Y	Y	Y	14	High
Tadess et al [43], 2017	Y	Y	Y	N	Y	Y	N	Y	Y	Y	Y	NA	Y	Y	12	High
Negussie et al [44], 2016	Y	Y	Y	N	Y	Y	Y	Y	Y	Y	Y	Y	Y	Y	14	High
Bosu et al [45], 1997	Y	Y	Y	N	Y	Y	Y	Y	Y	N	Y	N	Y	Y	14	High
Desgrées du Loû et al [46], 1994	Y	Y	Y	Y	Y	Y	N	Y	NC	N	Y	Y	Y	Y	10	Moderate
Sato R. [47], 2019	Y	Y	Y	N	Y	Y	N	NA	NA	N	Y	Y	Y	Y	10	Moderate
Akwataghibe, N. N. et al [48], 2019	Y	Y	Y	N	Y	Y	Y	Y	Y	Y	Y	Y	Y	Y	14	High
Yismaw, A. E. et al [49], 2019	Y	Y	Y	N	Y	Y	Y	Y	Y	Y	Y	Y	Y	Y	14	High
Ntenda P. [50], 2019	Y	Y	Y	N	Y	Y	N	Y	Y	Y	Y	Y	Y	Y	13	High
Okenwa, U. J. et al [51], 2019	Y	Y	Y	N	Y	Y	Y	Y	Y	Y	Y	Y	Y	Y	14	High
Mthiyane, T. N et al [52], 2019	Y	Y	Y	N	Y	Y	Y	Y	Y	Y	Y	Y	Y	Y	14	High
Mekonnen, A. G. et al [53], 2019	Y	Y	Y	N	Y	Y	Y	Y	Y	Y	Y	Y	Y	Y	14	High

Ibraheem, R. et al [54], 2019	Y	Y	Y	N	Y	Y	Y	Y	Y	Y	Y	Y	Y	Y	14	High

3. Full-text articles excluded with reasons (n=28)

No	Author, year	Title	Reason for exclusion
1	Aloysius R. 2016	Reasons for non-timely completion of the routine infant immunization schedule by children in rural South West Uganda	Letter to the editor
2	Bukenya et al, 1991	Possible reasons for non-completion of immunization in an urban settlement of Papua New Guinea.	Conducted in another region
3	Bender D et al, 1988	Immunization drop-outs and maternal behavior: evaluation of reasons given and strategies for maintaining gains made in the national vaccination campaign in Liberia	Full text not accessed
4	Adeloye et al, 2017	Coverage and determinants of childhood immunization in Nigeria: A systematic review and meta-analysis.	Review
5	Namuigi et al, 2005	Barriers to measles immunization: the beliefs and attitudes of caregivers in Goroka, Eastern Highlands Province, Papua New Guinea	Conducted in another region
6	Downing et al, 2018	Barriers to the delivery of the hepatitis B birth dose: a study of five Papua New Guinean hospitals in 2007.	Conducted in another region
7	Craciun et al, 2012	"Who will take the blame?": Understanding the reasons why Romanian mothers decline HPV vaccination for their daughters	Conducted in another region
8	Dammann et al, 1990	Vaccination--coverage of under-fives, validity of records, and the impact of mass campaigns in the Edendale/Vulindlela district of KwaZulu	Focusing on vaccination records validity
9	Sackou et al, 2012	Complete immunization coverage and reasons for non-vaccination in a periurban area of Abidjan	Full text not available in English
10	Douba et al, 2015	Sociodemographic factors associated with incomplete immunization of children aged 12 to 59 months in six West African countries	Full text not available in English
11	Nasiru et al,	Breaking community barriers to polio vaccination in northern Nigeria: the impact of a grass roots mobilization campaign (Majigi).	Focusing on impact of a grass roots mobilization
12	Sackou et al, 1990	Complete immunization coverage and reasons for non-vaccination in a periurban area of Abidjan	Full text not accessed
13	Mwamba et al, 2017	Vaccination coverage and factors influencing routine vaccination status in 12 high risk health zones in the Province of Kinshasa City, Democratic	supplement paper

		Republic of Congo (DRC), 2015	
14	Olorunsaiye et al, 2017	Missed opportunities and barriers for vaccination: a descriptive analysis of private and public health facilities in four African countries	Supplement paper
15	Michael et al, 2014	An Assessment of the Reasons for Oral Poliovirus Vaccine Refusals in Northern Nigeria	Supplement paper
16	Burnett et al., 2018	Impact of vaccine stock-outs on infant vaccination coverage: a hospital-based survey from South Africa	Focus on impact of vaccine stock-outs
17	Jalloh et al, 2019	Rapid behavioral assessment of barriers and opportunities to improve vaccination coverage among displaced Rohingyas in Bangladesh, January 2018	Conducted in another region
18	Koruk et al, 2013	A case of childhood vaccination barrier: migrant and seasonal farmworkers	Conducted in another region
19	Ports et al, 2013	Barriers and Facilitators to HPV Vaccination: Perspectives from Malawian Women	Focus on adult vaccines
20	Attia et al, 2018	On surmounting the barriers to HPV vaccination: we can do better	Focus on adult vaccines
21	Sato R. 2019	Barriers and facilitators of immunisation in refugees and migrants in Australia: an east-African case study	Conducted in another region
22	Carter, E. D., et al. 2019	Impact of vaccination delay on deaths averted by pneumococcal conjugate vaccine: Modeled effects in 8 country scenarios	Focus on impact of vaccination delay on deaths
23	Ferreras, E., et a.l 2016	Delayed second dose of oral cholera vaccine administered before high-risk period for cholera transmission: Cholera control strategy in Lusaka, 2016	Included adults
24	Hajizadeh M. et al 2019	Decomposing socioeconomic inequality in child vaccination in the Gambia, the Kyrgyz Republic and Namibia	Focus on socioeconomic inequalities
25	Ko, L. K. et al 2019	"We brought our culture here with us": A qualitative study of perceptions of HPV vaccine and vaccine uptake among East African immigrant mothers	Focus on adult vaccines
26	Madlon-Kay, D. J., & Smith, E. R. 2019	Interpreters' knowledge and perceptions of childhood vaccines: Effect of an educational session	Focus on effects of an educational session on interpreter beliefs and knowledge
27	Tambe, T. A. et al, 2019	Reasons for non-compliance to immunization among Fulani children aged between 0-11 months in the Vekovi community in Cameroon	Letter to the editor
28	Hvass, A. M. F. & Wejse, C. 2019	High coverage of polio immunization program in refugees resettling in Denmark. A cross-sectional study of polio serology in newly arrived refugees	Conducted in another region

References

1. **Aloysius R**. Reasons for non-timely completion of the routine infant immunization schedule by children in rural South West Uganda. *Canadian journal of public health. Revue canadienne de santé publique* January 2016 DOI: 10.17269/CJPH.106.5393
2. **Bukenya, GB and Freeman PA** Possible reasons for non-completion of immunization in an urban settlement of Papua New Guinea. *Papua and New Guinea medical journal. Vol.1.22-5.*1991-Mar
3. **Bender D and Macauley RJ** Immunization drop-outs and maternal behavior: evaluation of reasons given and strategies for maintaining gains made in the national vaccination campaign in Liberia. *International quarterly of community health education. Vol. 9 (*1988*)* 283-98
4. **Adeloye DJ, Wura A, Ann O, Ogundipe O, Mosaku O, Gadanya MA, Oni G,** Coverage and determinants of childhood immunization in Nigeria: A systematic review and meta-analysis. *Vaccine.* Vol. 35 (2017) 2871-2881
5. **Namuigi P, Phuanukoonnon S.** Barriers to measles immunization: the beliefs and attitudes of caregivers in Goroka, Eastern Highlands Province, Papua New Guinea. *Papua New Guinea Medical Journal* Vol. 48(2005) 183-187
6. **Downing SG, Lagani W, Guy R, Hellard M** Barriers to the delivery of the hepatitis B birth dose: a study of five Papua New Guinean hospitals in 2007. *Papua New Guinea Medical Journal.* Vol. 51 (2018) 47-55
7. **Craciun C, Baban A** "Who will take the blame?": Understanding the reasons why Romanian mothers decline HPV vaccination for their daughters. *Vaccine.* Vol. 30 (2012) 6789-6793
8. **Dammann DF, Solarsh GC, Patrick ME, Ijsselmuiden CB** Vaccination--coverage of under-fives, validity of records, and the impact of mass campaigns in the Edendale/Vulindlela district of KwaZulu. *South African medical journal.*Vol.78 (1990) 12:729-33
9. **Sackou KJ, Oga ASS, Desquith AA, Houenou Y, Kouadio KL.** Complete immunization coverage and reasons for non-vaccination in a periurban area of Abidjan. *Bulletin de la Societe de pathologie exotique* Vol. 105 (2012) 4: 284-90
10. **Douba A, Aka LB,Yao GH, Zengbe-Acray P, Akani B C, Konan NG.** Sociodemographic factors associated with incomplete immunization of children aged 12 to 59 months in six West African countries. *Sante Publique.* Vol.27(2015) 4:575-84
11. **Nasiru S, Aliyu, Gambo G, Gasasira AA, Muktar H, Zubair MM, Sunusi UW, Hassana N, Abdulsalami E, Samer S** Breaking community barriers to polio vaccination in northern Nigeria: the impact of a grass roots mobilization campaign (Majigi). *Pathogens and Global Health.* Vol. 106 (2012) 3:166-171
12. **Sackou K J, Oga ASS, Desquith AA, Houenou Y, Kouadio KL**. Complete immunization coverage and reasons for non-vaccination in a periurban area of Abidjan. *Bulletin de la Societe de pathologie exotique.* Vol. 105 (1990) 4: 284-90
13. **Mwamba GN, Yoloyolo N, Masembe Y, Nsambu MN, Nzuzi C, Tshekoya P, Dah B, Kaya G.** Vaccination coverage and factors influencing routine vaccination status in 12 high risk health zones in the Province of Kinshasa City, Democratic Republic of Congo (DRC), 2015. *The Pan African Medical Journal* Vol.27 (2017) 3: 7-7
14. **Olorunsaiye CZ, Langhamer MS, Wallace AS, Watkins ML** Missed opportunities and barriers for vaccination: a descriptive analysis of private and public health facilities in four African countries. *The Pan African medical journal.* Vol. 27 (2017)S3: 6-6
15. **Michael CA, Ogbuanu IU, Storms AD, Ohuabunwo CJ, Corkum M, Ashenafi S, Achari P, Biya O, Nguku P, Mahoney F.** An Assessment of the Reasons for Oral Poliovirus Vaccine Refusals in Northern Nigeria. *Journal of Infectious Diseases.* Vol.210 (2014) S125-S130

16. **Burnett RJ, Mmoledi G, Ngcobo NJ, Dochez C, Seheri LM, Mphahlele MJ**. Impact of vaccine stock-outs on infant vaccination coverage: a hospital-based survey from South Africa *International Health.* Vol. 10 (2018)5: 376-381
17. **Jalloh MF, Bennett SD, Alam D, Kouta P, Lourenco D, Alamgir M, Feldstein LR, Ehlman D. C, Abad, N, Kapil N, Vandenent, M, Conklin L, Wolff B.** Rapid behavioral assessment of barriers and opportunities to improve vaccination coverage among displaced Rohingyas in Bangladesh, January 2018. *Vaccine*. Vol.37 (2019)6:833-838
18. **Koruk I, Simsek Z, Koruk STA** Case of childhood vaccination barrier: migrant and seasonal farmworkers. *Turkish Journal of Medical Science. Vol. 43(2013)3:* 424-429
19. **Ports KA, Reddy DM, Rameshbabu, A.** Barriers and Facilitators to HPV Vaccination: Perspectives from Malawian Women. *Women & Healt. Vol. 53(2013) 6:* 630-645
20. **Attia AC, Wolf J, Nunez AE** On surmounting the barriers to HPV vaccination: we can do better. *Annals of Medicine*. Vol.50 (2018) 3:209-225
21. **Abdi, I., Menzies, R., & Seale, H. (2019).** Barriers and facilitators of immunisation in refugees and migrants in Australia: an east-African case study. *Vaccine,* 37(44), 6724–6729. doi:10.1016/j.vaccine.2019.09.025
22. **Carter, E. D., Tam, Y., & Walker, N.** (2019). Impact of vaccination delay on deaths averted by pneumococcal conjugate vaccine: Modeled effects in 8 country scenarios. *Vaccine*, 37(36), 5242–5249. doi:10.1016/j.vaccine.2019.07.063
23. **Ferreras, E., Matapo, B., Chizema-Kawesha, E., Chewe, O., Mzyece, H., Blake, A., Pezzoli, L.** (2019). Delayed second dose of oral cholera vaccine administered before high-risk period for cholera transmission: Cholera control strategy in Lusaka, 2016. *PloS one*, 14(8), e0219040. doi:10.1371/journal.pone.0219040
24. **Hajizadeh M**. (2019). Decomposing socioeconomic inequality in child vaccination in the Gambia, the Kyrgyz Republic and Namibia, *Vaccine*, 37(44), 6609–6616. doi:10.1016/j.vaccine.2019.09.054
25. **Ko, L. K., Taylor, V. M., Mohamed, F. B., Do, H. H., Gebeyaw, F. A., Ibrahim, A., … Winer, R. L.** (2019). "We brought our culture here with us": A qualitative study of perceptions of HPV vaccine and vaccine uptake among East African immigrant mothers. *Papillomavirus research* (Amsterdam, Netherlands), 7, 21–25. doi:10.1016/j.pvr.2018.12.003
26. **Madlon-Kay, D. J., & Smith, E. R.** (2019). Interpreters' knowledge and perceptions of childhood vaccines: Effect of an educational session. *Vaccine*, S0264-410X(19)31517-8. Advance online publication. doi:10.1016/j.vaccine.2019.11.010
27. **Tambe, T. A., Tchetnya, X., Nkfusai, C. N., Shirinde, J., & Cumber, S. N.** (2019). Reasons for non-compliance to immunization among Fulani children aged between 0-11 months in the Vekovi community in Cameroon. *The Pan African medical journal*, *33*, 278. doi:10.11604/pamj.2019.33.278.16900
28. **Hvass, A. M. F. Wejse, C**. (2019). High coverage of polio immunization program in refugees resettling in Denmark. A cross-sectional study of polio serology in newly arrived refugees. *Expert Review of Vaccines,18.12,* 1317-1322 doi: 10.1080/14760584.2019.1698953

SPRINGER NATURE

Change of authorship request form - Journals (pre-acceptance)

Section 1: Please provide the current title of manuscript

Manuscript ID no.: PUBH-D-19-04480R1

Title: BARRIERS TO CHILDHOOD IMMUNIZATION IN SUB-SAHARAN AFRICA: A SYSTEMATIC REVIEW

Section 2: Please provide the previous authorship, in the order shown on the manuscript before the changes were introduced. Please indicate the corresponding author by adding (CA) behind the name.

	First name(s)	Family name	ORCID or SCOPUS id, if available.
1st author	JOSEPH BENJAMIN	BANGURA	ORCID: 0000-0003-0042-7226
2nd author	SHILI YUAN	XIAO (CA)	
3rd author	LEI	CHEN	
4th author	DAN	QIU	
5th author	FEIYUN	OUYANG	
6th author			
7th author			
8th author			
9th author			
10th author			

Please use an additional sheet if there are more than 10 authors.

SPRINGER NATURE Springer Nature is one of the world's leading global research, educational and professional publishers, created in May 2015 through the combination of Nature Publishing Group, Palgrave Macmillan, Macmillan Education and Springer Science+Business Media.

SPRINGER NATURE Change of authorship request form - Journals (pre-acceptance)

Section 3: Please provide a justification for change. Please use this section to explain your reasons for changing the authorship of your manuscript, e.g. what necessitated the change in authorship? Please refer to the (journal) policy pages for more information about authorship. Please explain why omitted authors were not originally included and/or why authors were removed on the submitted manuscript.

We did not remove or add new author. We rearranged the list, i.e. LEI CHEN moved from 3rd to 5th position. LEI CHEN was instrumental in the supervision and revision of the final manuscript.

Section 4: Proposed new authorship. Please provide your new authorship list in the order you would like it to appear on the manuscript. Please indicate the corresponding author by adding (CA) behind the name. If the Corresponding Author has changed, please indicate the reason under section 3.

	First name(s)	Family name (this name will appear in full on the final publication and will be searchable in various abstract and indexing databases)	Affiliated institute	E-mail address
1st author	JOSEPH BENJAMIN	BANGURA	XIANGYA SCHOOL OF PUBLIC HEALTH, CSU.	Jbangura64@yahoo.com
2nd author	SHUIYUAN	XIAO (CA)	XIANGYA SCHOOL OF PUBLIC HEALTH, CSU.	Shuiyuanxiao01503@163.com
3rd author	DAN	QIU	XIANGYA SCHOOL OF PUBLIC HEALTH, CSU.	166911058@csu.edu.cn
4th author	FEIYUN	OUYANG	XIANGYA SCHOOL OF PUBLIC HEALTH, CSU.	feiyun0716@foxmail.com
5th author	LEI	CHEN	YALE UNIVERSITY SCHOOL OF MEDICINE	lei.chen@yale.edu
6th author				
7th author				
8th author				
9th author				
10th author				

Please use an additional sheet if there are more than 10 authors.

SPRINGER NATURE Springer Nature is one of the world's leading global research, educational and professional publishers, created in May 2015 through the combination of Nature Publishing Group, Palgrave Macmillan, Macmillan Education and Springer Science+Business Media.

SPRINGER NATURE Change of authorship request form - Journals (pre-acceptance)

Section 5: Author contribution, Acknowledgement and Disclosures. Please use this section to provide a new disclosure statement and, if appropriate, acknowledge any contributors who have been removed as authors and ensure you state what contribution any new authors made (if applicable per the journal or book (series) policy). Please ensure these are updated in your manuscript - after approval of the change(s) - as our production department will not transfer the information in this form to your manuscript.

New acknowledgements:

NOT APPLICABLE

New Disclosures (financial and non-financial interests, funding):

NOT APPLICABLE

New Author Contributions statement (if applicable per the journal policy):

NOT APPLICABLE

State 'Not applicable' if there are no new authors.

SPRINGER NATURE Springer Nature is one of the world's leading global research, educational and professional publishers, created in May 2015 through the combination of Nature Publishing Group, Palgrave Macmillan, Macmillan Education and Springer Science+Business Media.

SPRINGER NATURE

Change of authorship request form - Journals (pre-acceptance)

Section 6: Declaration of agreement. *All* **authors, unchanged, new and removed** ***must*** **sign this declaration.**
(NB: Please print the form, (do)sign and return/upload a scanned copy. Please note that signatures that have been inserted as an image file are acceptable as long as it is handwritten. Typed names in the signature box are unacceptable.) * **Please delete as appropriate. Delete all of the bold if you were on the original authorship list and are remaining as an author.**

	First name	Family name		Signature	Date
1st author	JOSEPH BENJAMIN	BANGURA	I agree to the proposed new authorship shown in section 4 /an~~d the addition~~/remo~~val*of my~~ ~~name to the~~ auth~~orship~~ list /and the proposed change in corresponding author	[illegible]	9-03-2020
2nd author	SHUIYUAN	XIAO (CA)	I agree to the proposed new authorship shown in section 4 /an~~d the addition~~/r~~emoval*of~~ my ~~name to the~~ auth~~orship list~~ /and the proposed change in corresponding author	Shuiyuan Xiao	14-3-2020
3rd author	DAN	QIU	I agree to the proposed new authorship shown in section 4 /and ~~the addition~~/re~~moval*of my~~ ~~name to the auth~~orship list /and the proposed change in corresponding author	Dan Qiu	16-3-2020
4th authors	FEI YUN	OUYANG	I agree to the proposed new authorship shown in section 4 /and ~~the addition~~/re~~moval*of~~ my ~~name to the auth~~orship list /and the proposed change in corresponding author	Feixun Ouyang	11-3-2020
5th author	LEI	CHEN	I agree to the proposed new authorship shown in section 4 /and ~~the addition~~/re~~moval*of my~~ ~~name to th~~e author~~ship list~~ /and the proposed change in corresponding author	Lei Chen	11-3-2020
6th author			I agree to the proposed new authorship shown in section 4 /**and the addition/removal*of my name to the authorship list** /and the proposed change in corresponding author		
7th author			I agree to the proposed new authorship shown in section 4 /**and the addition/removal*of my name to the authorship list** /and the proposed change in corresponding author		

SPRINGER NATURE Springer Nature is one of the world's leading global research, educational and professional publishers, created in May 2015 through the combination of Nature Publishing Group, Palgrave Macmillan, Macmillan Education and Springer Science+Business Media.
